# Ferroptosis-inducing agents compromise in vitro human islet viability and function

**DOI:** 10.1038/s41419-018-0506-0

**Published:** 2018-05-22

**Authors:** Antonio Bruni, Andrew R. Pepper, Rena L. Pawlick, Boris Gala-Lopez, Anissa F. Gamble, Tatsuya Kin, Karen Seeberger, Gregory S. Korbutt, Stefan R. Bornstein, Andreas Linkermann, A. M. James Shapiro

**Affiliations:** 1 Clinical Islet Transplant Program, Alberta Diabetes Institute, University of Alberta, Edmonton, AB Canada; 2 Department of Surgery, University of Alberta, Edmonton, AB Canada; 3 QEII Health Science Centre, Dalhousie University, Halifax, NS Canada; 4 Clinic for Internal Medicine 3, Hospital Carl Gustav Carus Dresden, Dresden, Germany; 5 Division of Nephrology, Clinic for Internal Medicine 3, Hospital Carl Gustav Carus Dresden, Dresden, Germany

## Abstract

Human islet transplantation has been hampered by donor cell death associated with the islet preparation procedure before transplantation. Regulated necrosis pathways are biochemically and morphologically distinct from apoptosis. Recently, ferroptosis was identified as a non-apoptotic form of iron-dependent regulated necrosis implicated in various pathological conditions. Mediators of islet oxidative stress, including glutathione peroxidase-4 (GPX4), have been identified as inhibitors of ferroptosis, and mechanisms that affect GPX4 function can impact islet function and viability. Ferroptosis has not been investigated directly in human islets, and its relevance in islet transplantation remains unknown. Herein, we sought to determine whether in vitro human islet viability and function is compromised in the presence of two distinct ferroptosis-inducing agents (FIA), erastin or RSL3, and whether these effects could be rescued with ferroptosis inhibitors, ferrostatin-1 (Fer-1), or desferrioxamine (DFO). Viability, as assessed by lactate dehydrogenase (LDH) release, revealed significant death in erastin- and RSL3-treated islets, 20.3% ± 3.8 and 24.4% ± 2.5, 24 h post culture, respectively. These effects were ameliorated in islets pre-treated with Fer-1 or the iron chelator, desferrioxamine (DFO). Stimulation index, a marker of islet function revealed a significant reduction in function in erastin-treated islets (control 1.97 ± 0.13 vs. 50 μM erastin 1.32 ± 0.1) (*p* < 0.05). Fer-1 and DFO pre-treatment alone did not augment islet viability or function. Pre-treatment of islets with erastin or Fer-1 did not impact in vivo engraftment in an immunodeficient mouse transplant model. Our data reveal that islets are indeed susceptible to ferroptosis in vitro, and induction of this novel cell death modality leads to compromised islet function, which can be recoverable in the presence of the ferroptosis inhibitors. The in vivo impact of this pathway in islet transplantation remains elusive given the constraints of our study, but warrants continued investigation.

## Introduction

The inception of the “Edmonton Protocol” by Shapiro and colleagues, and more recent modifications and improvements were critical in establishing islet transplantation as a viable therapeutic option for select patients with type 1 diabetes mellitus^1, 2^. With complete insulin-independence up to 1 year post transplant, 5-year follow-up of early transplant recipients demonstrated maintained graft function with presence of C-peptide, correction of hemoglobin A1C, and stabilization of glycemic control, but the majority returned to modest exogenous insulin therapy over time^3^. Early insights into long-term success rates suggest that there are numerous limitations associated with engraftment outcomes, many of which occur during islet isolation and in the acute and peri-transplant period.

When transplanted into the portal vein, it is estimated that up to 70% of the transplanted islet mass is lost in the acute and peri-transplant period, resulting from numerous factors. Such factors include the instant blood-mediated inflammatory reaction, hypoxia, delayed revascularization, and inflammatory cytokines^4^. These events stimulate the initiation of cell death cascades, apoptosis, and necrosis, contributing to islet loss during the preparation procedure and within hours and days of transplant, long before the initiation of alloimmune or recurrent autoimmune responses^5–7^. In contrast to non-immunogenic apoptosis, necrosis is increasingly recognized as the most potent trigger of the immune system^8^. Likely, this event is further exaggerated by human leukocyte antigen (HLA) or species incompatibility^9–11^. Strategies to deter early cell death could critically augment islet engraftment thereby improving long-term graft function. Along these lines, strategies to prevent caspase-dependent islet death, including the administration of interleukin-1β receptor agonists^12^, withaferin A^13^, and caspase-specific inhibitors both in vitro and in vivo have been explored previously^7, 14–16^.

Non-apoptotic cell death has been identified in various pathological conditions, including myocardial infarction, stroke, ischemia-reperfusion injury, and many others^17^. In contrast to unregulated necrosis, whereby cell death can occur through spontaneous, “accidental” triggers like trauma, regulated necrosis occurs through distinct biochemical mediators that activate molecular machinery^8^. One particular subroutine of regulated necrosis, termed ferroptosis, is morphologically and biochemically distinct from other forms of cell death, that it is iron-dependent and non-apoptotic^18, 19^. Ferroptosis was first described in parallel to the identification ferrostatin-1 (Fer-1), an inhibitor of this cell death pathway that functioned to prevent erastin-induced cell death^8, 9, 17, 18^. Erastin, a small potent molecule capable of selectively inhibiting the X_c_-cystine/glutamate antiporter required for glutathione (GSH) biosynthesis, induces ferroptosis^17, 18^. Subsequent to intracellular GSH depletion, the GSH-dependent, lipid repair enzyme, glutathione peroxidase 4 (GPX4), lacks the ability to sufficiently repair aberrant downstream accumulation of reactive oxygen species (ROS)^17, 18, 20–22^. RSL3, has recently been identified as a potent GPX4-specific inhibitor and known inducing agent of ferroptosis^18, 21^. Relative to other native tissues, islets exhibit reduced antioxidant defences, and as a result are susceptible to the dysregulation of free radical production and subsequent oxidative stress^23, 24^. Desferrioxamine (DFO), an iron chelator has demonstrated some benefit to improving islet viability. Ferrostatins were previously demonstrated to reduce ferroptosis-induced death in cellular models of Huntington’s disease, periventricular leukomalacia, kidney tubular necrosis, and acute kidney injury^17, 25^. Given that key mediators of islet survivability are also key targets that induce ferroptosis, it has yet to be elucidated if islets are susceptible to ferroptosis-induced cell death.

Herein, we sought to establish whether human islets exhibit reduced islet viability and function when challenged with ferroptosis-inducing agents (FIAs), erastin, or RSL3, in vitro. We also sought to determine whether inhibitors of ferroptosis such as the small molecule Fer-1 could rescue human islets from the subsequent deleterious effects of these agents. Given the conceived cytoprotective effects of Fer-1 in other disease models, we evaluated whether pre-conditioning with Fer-1 could augment islet engraftment in an immunodeficient, marginal human islet transplant model, as well as assess whether in vitro challenge with erastin could compromise subsequent in vivo engraftment in a full-dose transplant model.

## Results

### Human islets cultured in the presence of erastin exhibit impaired function and viability

**Fig. 1:**
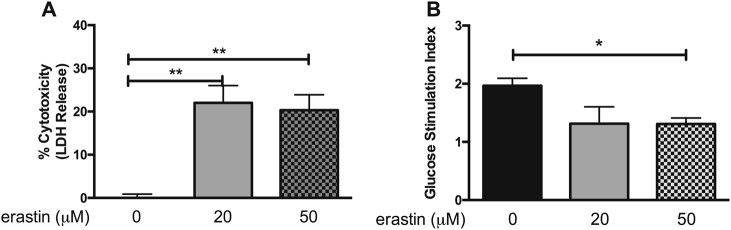
In vitro human islet viability and function is compromised in the presence of erastin. **a** Human islets challenged with 20 and 50 μM erastin exhibit increased LDH release relative to non-treated control islets (*p* < 0.01). Data represented as percent of control. **b** Erastin treatment impairs glucose-stimulated insulin secretion in human islets. (*p* < 0.05). Data represented as mean ± SEM. (One-way ANOVA followed by Tukey’s multiple comparison test). Triplicate samples from at least three human pancreata

Cell-free supernatants collected 24 h post culture revealed significant levels of LDH from islets challenged with 20 or 50 μM erastin compared to supernatants from islet cultured in CMRL alone (Fig. 1a) (20 μM erastin: 22.0% ± 4.0; 50 μM erastin: 20.3% ± 3.8, *p* < 0.01). When assessed for islet function, human islets exposed to 50 μM erastin exhibited significantly reduced insulin-secreting capacity in response to glucose vs. control islets (stimulation index: control 1.97 ± 0.13 vs. 50 μM erastin 1.32 ± 0.1, *p* < 0.05) (Fig. 1b). These results indicated that islets are sensitive to ferroptosis.

### Pre-treatment with 1 μM Fer-1 and 100 μM DFO rescues erastin-induced islet cell death

**Fig. 2:**
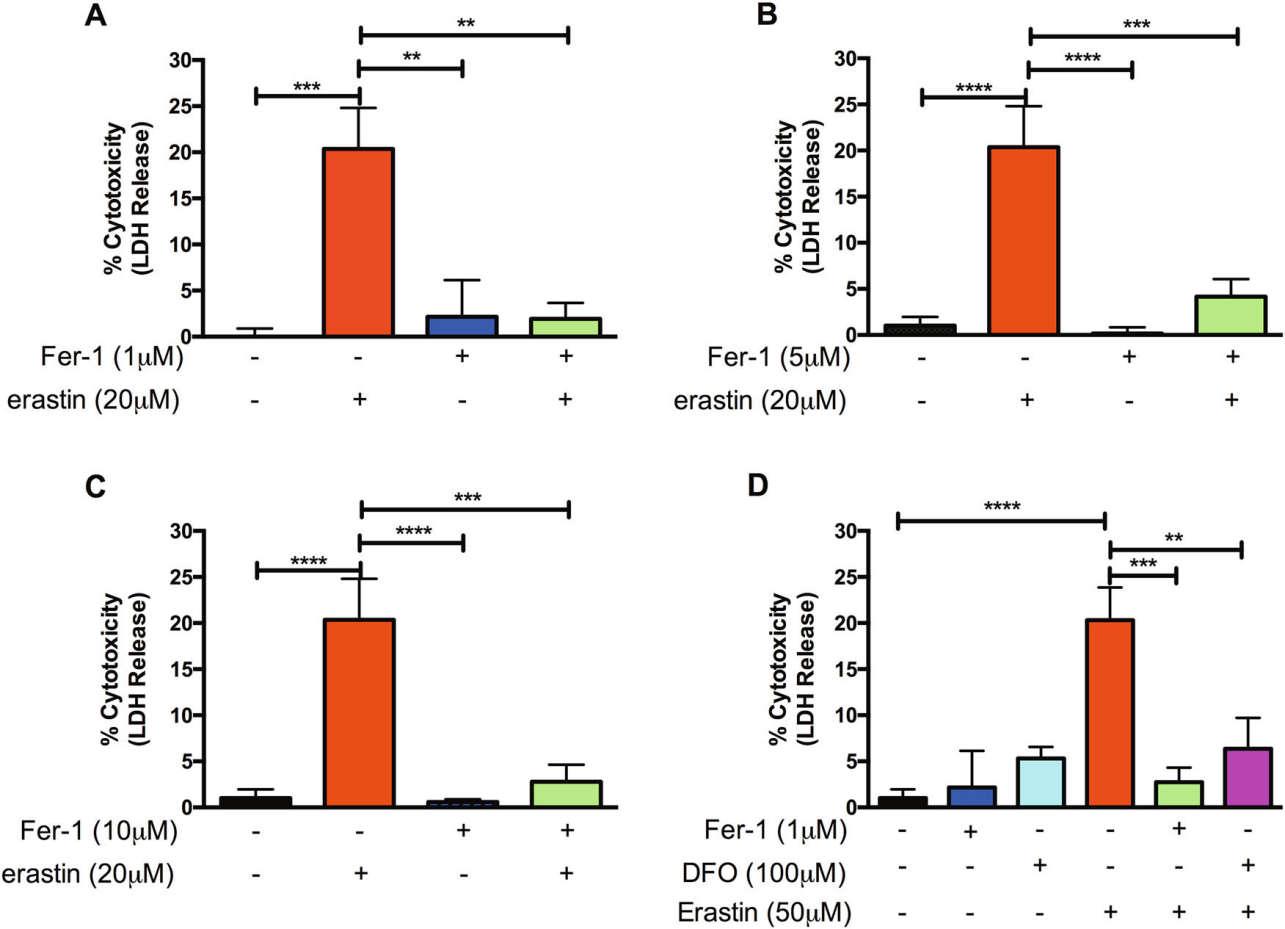
Assessment of LDH release of human islets pre-treated with or without Fer-1 or DFO in the presence of erastin. **a**–**c** Human islets exhibit significantly reduced islet cell death when pre-treated for 24 h with 1, 5, or 10 μM Fer-1 and subsequently challenged with erastin for an additional 24 h. **p < 0.01, ***p < 0.001, ****p < 0.0001 (one-way ANOVA). **d** 1 μM Fer-1 and 100 μM DFO exhibited reduced LDH release in the presence of 50 μM Fer-1 erastin relative to erastin-alone-treated islets. **p < 0.01, ***p < 0.001, ****p < 0.0001 (one-way ANOVA). Data represented as mean ± SEM, Triplicate samples from at least three human pancreata

After 24 h of 20 μM erastin treatment, human islets pre-treated with 1, 5, or 10 μM Fer-1 exhibited significantly reduced LDH levels as compared to islets treated with erastin alone (*p* < 0.01, *p* < 0.001, and *p* < 0.001, respectively) (Fig. 2a–c). Notably, in all cases, islets pre-treated with Fer-1 exhibited no significant difference in LDH release compared to their erastin-treated counterparts. Moreover, in the presence of 50 μM erastin, 24-h pre-treatment with 1 μM Fer-1 or 100 μM DFO exhibited significant cytoprotection compared to erastin treatment alone (*p* < 0.001 and *p* < 0.01, respectively) (Fig. 2d). Similarly, islets pre-treated with 5 or 10 μM Fer-1 conferred significant cytoprotection in the presence of 50 μM erastin as exhibited by reduced LDH release (data not shown). These results indicate that ferroptosis in islets can be prevented by DFO and Fer-1.

### Fer-1 pre-treatment preserves sGSIS in the presence of erastin

**Fig. 3:**
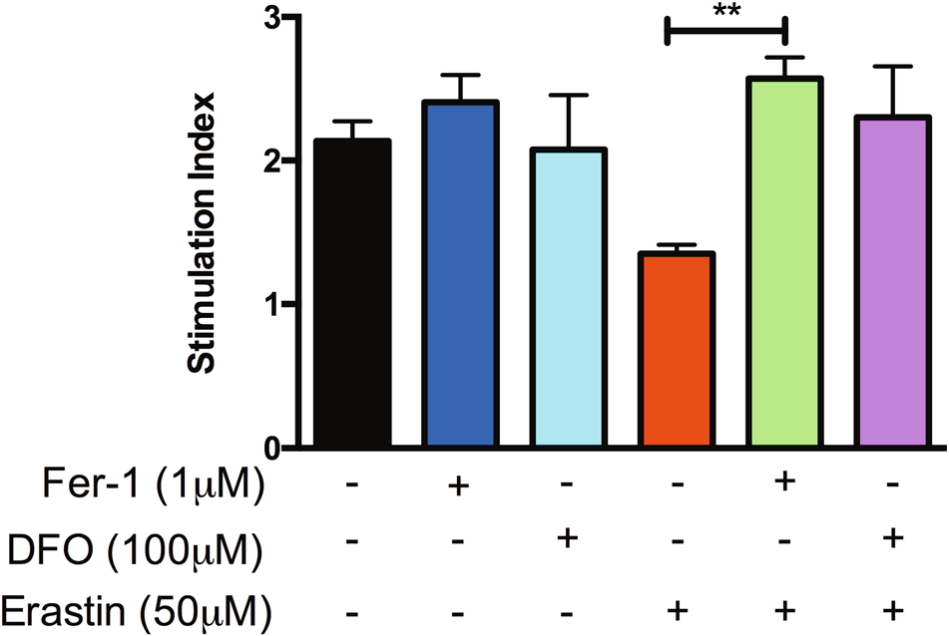
Fer-1 pre-treatment maintains sGSIS in human islets treated with erastin. Human islets were pre-treated ±1 μM Fer-1 or ±100 μM DFO for 24 h and subsequently challenged ±50 μM erastin for an additional 24 h. Subsequent to culture, islets were collected and assessed for sGSIS. Cell-free supernatants were assessed for insulin secretion via ELISA and expressed as stimulation index (insulin secreted in response to 16.7 mM glucose vs. insulin secreted in response to 2.8 mM glucose). Data represented as mean ± SEM, triplicate samples from at least three human pancreata. **p < 0.01 (one-way ANOVA, followed by Tukey’s multiple comparison test)

Since human islets treated with 50 μM erastin exhibited significant impairment of glucose-stimulated insulin secretion relative to non-treated control islets, we evaluated whether pre-treatment with 1 μM Fer-1 or 100 μM DFO could improve glucose-stimulated insulin secretion (GSIS) in the presence of erastin. Human islets pre-treatment with 1 μM Fer-1 followed by erastin treatment preserved the insulin-secreting capacity of human islets (stimulation index 1 μM Fer-1 + 50 μM erastin: 2.29 ± 0.06), whereas islets treated with erastin alone exhibited reduced insulin-secreting capabilities (stimulation index 50 μM erastin: 1.35 ± 0.1) (*p* < 0.01) (Fig. 3). Pre-treatment of islets with DFO followed by erastin treatment exhibited a non-significant preservation in insulin-secreting capacity (stimulation index 100 μM DFO + 50 μM erastin: 2.3 ± 0.36; *p* > 0.05). As anticipated, Fer-1 alone and DFO alone-treated islets demonstrated similar insulin secretory capacity as control islets (stimulation index control: 2.1 ± 0.14 vs. 1 μM Fer-1: 2.41 ± 0.19 vs. 100 μM DFO: 2.1 ± 0.38, *p* > 0.05) (Fig. 3). These data demonstrate the functional relevance of ferroptosis in islets with respect to sGSIS.

### RSL3 compromises human islet viability and moderately impairs sGSIS

**Fig. 4:**
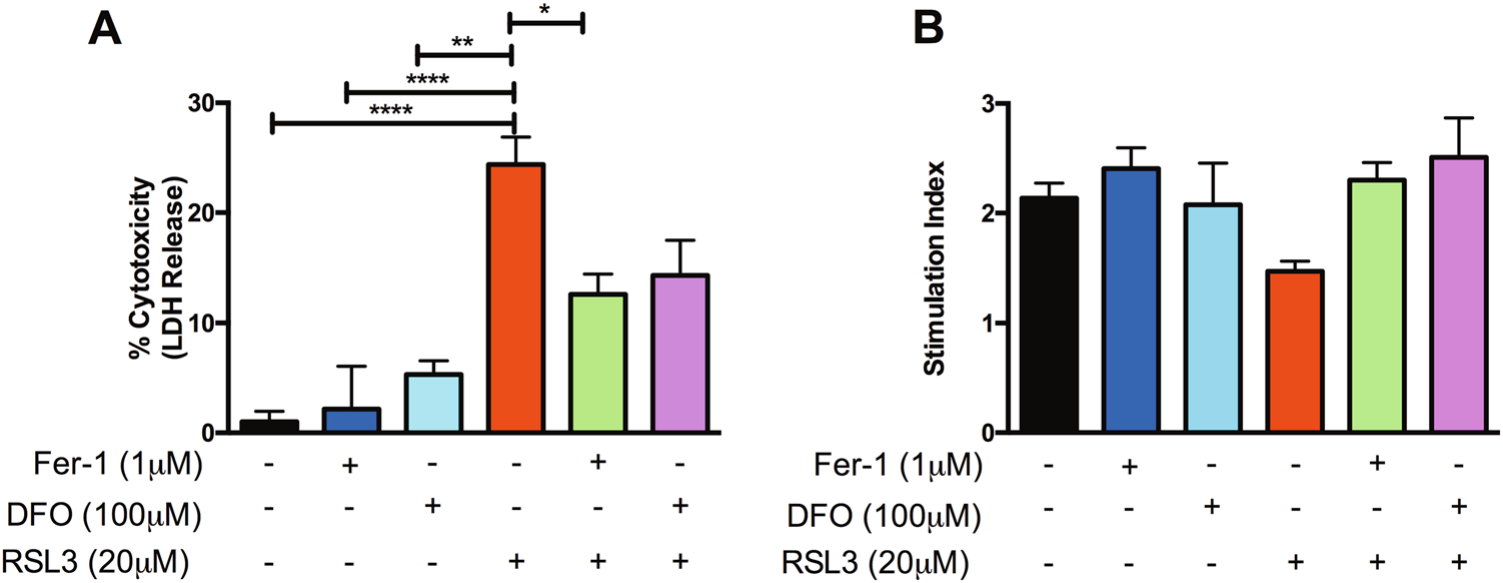
Evaluation of in vitro human islet viability and function in the presence of the ferroptosis-inducing agent, RSL3. Human islets were pre-treated with or without 1 μM Fer-1 for 24 h and subsequently challenged with or without 20 μM RSL3 for an additional 24 h. **a** Human islets exhibit significantly increased cell death as assessed by LDH in the presence of 20 μM RSL3. Pre-treatment with 1 μM Fer-1 or 100 μM DFO preserves islet viability in the presence of RSL3. *p < 0.05, **p < 0.01, ***p < 0.001, ****p < 0.0001 (one-way ANOVA). **b** sGSIS evaluation of the insulin-secreting capacity of human islets cultured alone, pre-conditioned with 1 μM Fer-1 or 100 μM DFO in the presence of 20 μM RSL3. Cell-free supernatants were assessed for insulin secretion via ELISA and expressed as stimulation index. Data represented as mean ± SEM, triplicate samples from at least three human pancreata

To further elucidate whether FIAs could compromise in vitro viability and function, human islets were cultured for 24 h in the presence of an alternative known stimulator of ferroptosis, RSL3, at 20 μM concentration subsequent to 24 h of pre-conditioning with or without 1 μM Fer-1 or 100 μM DFO. Cell-free supernatants from islets cultured in RSL3 alone exhibited significantly elevated LDH in comparison to non-treated control islets alone (control: 1.7% ± 0.1 vs. 20 μM RSL3: 24.4% ± 2.5, *p* < 0.0001). Islets pre-treated with 1 μM Fer-1 and subsequently challenged with 20 μM RSL3 for an additional 24 h exhibited significantly reduced cell death in comparison to RSL3 treatment alone (20 μM RSL3: 24.4% ± 2.5 vs. 1 μM Fer-1 ± 20 μM RSL3: 12.57% ± 1.85, *p* < 0.05) (Fig. 4a). Islets pre-treated with 100 μM DFO and subsequently challenged with 20 μM RSL3 exhibited reduced, albeit non-significant reduction in LDH release relative to RSL3 treatment alone (20 μM RSL3: 24.4% ± 2.5 vs. 100 μM DFO ± 20 μM RSL3: 14.28% ± 3.2, *p* > 0.05) (Fig. 4a). When evaluating the insulin-secreting capacity of islets through sGSIS, islets challenged with 20 μM RSL3 were not significantly different from control islets (control: 2.35 ± 0.07 vs. 20 μM RSL3: 1.80 ± 0.23, *p* > 0.05). These results indicated that ferroptosis in human islets can be induced by type 1 (erastin) and type 2 (RSL3) ferroptosis inducers. Induction of either type of FIAs is sensitive to DFO and Fer-1.

### Erastin, but not RSL3, treatment significantly increases CHAC1 mRNA expression in human islets

**Fig. 5:**
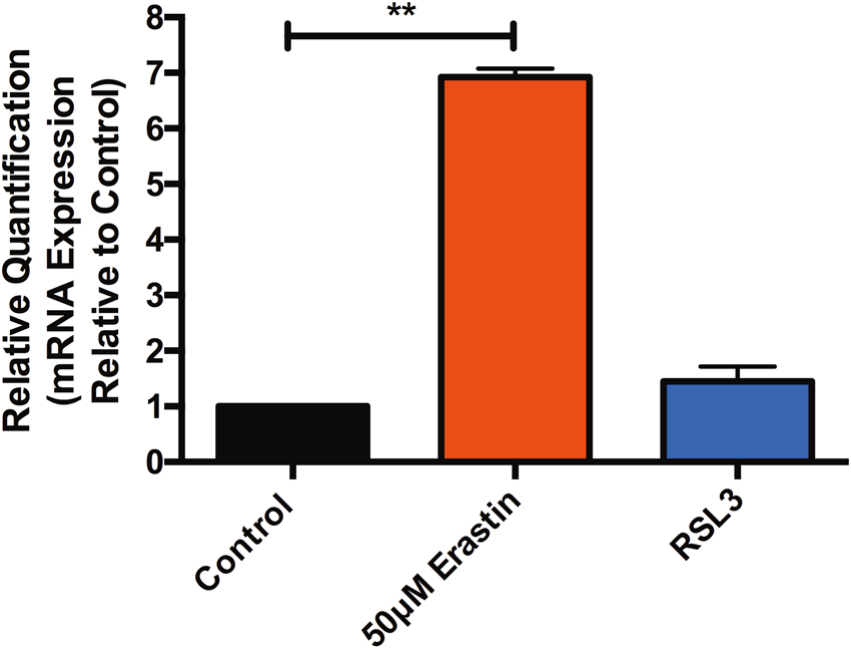
Erastin, but not RSL3 treatment augments *CHAC1* mRNA expression in human islets. Human islets treated for 24 h in the presence of 50 μM erastin exhibit a significant increase in the glutathione-depleting gene, *CHAC1* (**p < 0.01). In contrast, 20 μM RSL3 treatment did not significantly elevate *CHAC1* expression relative to non-treated control islets

Twenty-four hours post treatment with 50 μM erastin or 20 μM RSL3, human islets were collected and assessed for messenger RNA (mRNA) expression of the glutathione-depleting gene, *CHAC1*. Relative to non-treated control islets, erastin-treated islets exhibited a significant upregulation of mRNA expression of *CHAC1* (*p* < 0.01). In contrast, RSL3-treated islets revealed no significant difference in *CHAC1* expression relative to non-treated islets (*p* > 0.05) (Fig. 5). In these experiments, we describe an easily detectable marker to differentiate between GSH depletion-induced ferroptosis and direct GPX4 inhibition.

### Engraftment efficacy of Fer-1-treated human islets transplanted under the renal capsule of immunodeficient mice

**Fig. 6:**
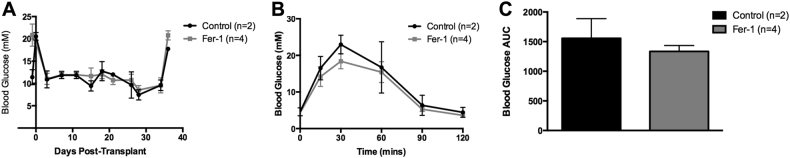
Efficacy of marginal dose human islet transplants under the renal capsule of C57BL/6 RAG^−/−^ recipients. **a** Non-fasting blood glucose measurements of euglycemic recipients post transplant. Recipients of control and 1 μM Fer-1 marginal (500 IEQs) human islets exhibited robust glycemic control until graft retrieval (arrow). **b** Blood glucose prolife post-dextrose bolus of control (black, *n* = 2) and 1 μM Fer-1 (gray, *n* = 4). **c** Blood glucose area under the curve (AUC) analysis did not differ between control and 1 μM Fer-1-treated islet recipients. Mice were administered 3 mg/kg 25% dextrose i.p. Blood glucose measurements were monitored at *t* = 0, 15, 30, 60, 90, and 120 min

Islet engraftment efficacy of human islets pre-treated with or without Fer-1 for 48 h was evaluated in a marginal islet transplant mass model (500 IEQs per recipient, *n* = 2 control, *n* = 4 Fer-1). All recipients became euglycemic subsequent to transplant in both transplant groups. Daily non-fasting blood glucose monitoring of euglycemic transplant recipients revealed no difference between control and Fer-1-treated islet recipients (Fig. 6a). Intraperitoneal glucose tolerance tests (IPGTTs) were performed on all marginal islet transplant, euglycemic recipients 35 days post transplant. Mice in both transplant groups exhibited a physiological response to glucose bolus, with a prompt restoration of normoglycemia up to 120 min post-dextrose infusion (Fig. 6b). Furthermore, there was no significant difference in mean area under the curve (AUC) ± SEM (AUC control: 1557 ± 300.4 mmol/L/120 min vs. Fer-1: 1335 ± 100.1 mmol/L/120 min, *p* > 0.05, unpaired *t* test, Fig. 6c). This experiment suggests that pre-treatment of islets with the ferroptosis inhibitor, Fer-1, does not enhance engraftment in this transplant model. However, it cannot be excluded that more stable second generation ferrostatins may still be beneficial.

### Human islets pre-treated with erastin do not exhibit impaired engraftment

**Fig. 7:**
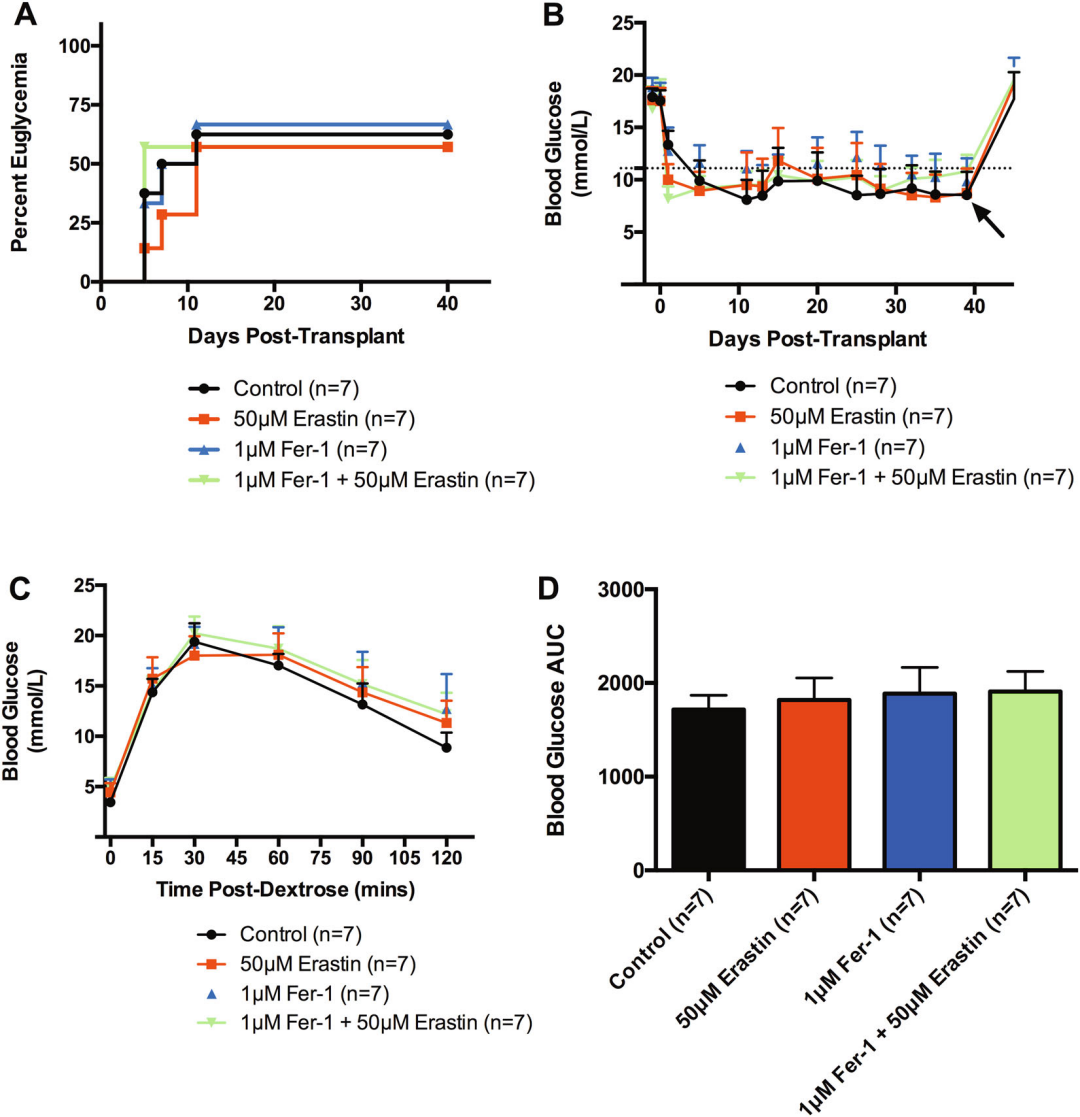
Efficacy of full-dose (1500 IEQs) human islet transplants under the renal capsule of C57BL/6 RAG^−/−^ recipients. **a** Percent euglycemia of full-dose (1500 IEQs) human islet transplant recipients receiving islets cultured alone (control, *n* = 7), or challenged with 50 μM erastin (red, *n* = 7), 1 μM Fer-1 alone (blue, *n* = 7), or 1 μM Fer-1 ± 50 μM erastin (green, *n* = 7). **b** Non-fasting blood glucose measurements of human islet recipients post transplant. Euglycemic recipients maintained glycemic control throughout the duration of engraftment until graft retrieval (arrow). Dotted line exhibits maximum threshold of normoglycemia (≤11.1 mmol/L). Data represented as mean ± SEM per group. **c** Blood glucose profile post-dextrose bolus of control (black, *n* = 7), 20 μM erastin (red, *n* = 7), 1 μM Fer-1 alone (blue, *n* = 7), or 1 μM Fer-1 ± 50 μM erastin (green, *n* = 7). **d** Mean blood glucose area under the curve (AUC). Mice were administered 3 mg/kg 25% dextrose i.p. Blood glucose measurements were monitored at *t* = 0, 15, 30, 60, 90, and 120 min. Data represented as mean ± SEM per group

Transplant recipients of human islets pre-treated for 24 h with or without 1 μM Fer-1 and additional 24 h challenge with or without 50 μM erastin revealed no significant difference in restoration of euglycemia between all transplant groups (Fig. 7a). Non-fasting, mean blood glucose profiles of all four transplant groups revealed similar glycemic trends (Fig. 7b). Recipients in all groups reverted back to hyperglycemia upon graft recovery nephrectomy, thus confirming graft-dependent euglycemia. To evaluate in vivo graft function, IPGTT was performed on transplant recipients 40 days post transplant. Islet transplant recipients from all groups revealed similar blood glucose profiles post-dextrose infusion and exhibited a restoration of normoglycemia within 120 min (Fig. 7c). No discernable difference in blood glucose mean AUC was observed between groups (Fig. 7d). These data suggest that islet engraftment is not compromised subsequent to erastin pre-treatment in this transplant scenario.

## Discussion

Strategies to ameliorate early islet loss in culture and in the acute transplant period may positively contribute to long-term engraftment outcomes. During organ procurement, islet isolation, and transplantation, islets experience considerable oxidative stress that contributes to islet injury and loss. GSH has been identified as an important intracellular antioxidant capable of mitigating the accumulation of ROS. Erastin has demonstrated the ability to induce ferroptosis by minimizing cystine uptake, reducing intracellular GSH levels, thus contributing to ferroptosis-induced cell death in various disease models^17^. Moreover, the GPX4 inhibitor, RSL3 has also been identified as an additional inducing agent of ferroptosis. Given the importance of GSH and GPX4 in islet viability and function, the role of ferroptosis has yet to be elucidated in islet transplantation.

The inability to resolve accumulating ROS results in oxidative stress. It has previously been demonstrated that considerable ROS is generated during isolation and transplantation^26, 27^. Prolonged oxidative stress has been associated with compromised islet viability and function^28^. As such, host antioxidant systems play an integral role in reducing excessive ROS thus minimizing cellular damage and impairment. Islets exhibit reduced intrinsic antioxidant enzyme expression and activity relative to other host tissues and in an effort to preserve islet function, the administration of exogenous antioxidants has provided substantial cytoprotective benefit^23, 24, 29^. GSH, a tri-peptide synthesized from glutamate, cysteine, and glycine has been established as an important intracellular antioxidant^30^. The exogenous administration of glutamine, which contributes to glutamate synthesis, a GSH precursors, have exhibited improved islet function^31, 32^. Intra-ductal administration of glutathione precursors, such as l-glutamine, have previously demonstrated the ability to augment intracellular glutathione pools, and reduce oxidative injury during human pancreatic islet isolation^32^. Disruption of the cell’s glutamine-synthesizing capacity may alternatively contribute to the cell’s demise.

In the present study, we evaluated whether human islet viability and function could be compromised in the presence of FIAs, erastin, and RSL3. Our results revealed that erastin and RSL3 exacerbated LDH release, indicative of islet cell ferroptosis. Erastin exhibited the ability to impair the insulin-secreting capacity of islets when assessed via sGSIS, and for islets pre-conditioned with Fer-1 (or DFO), the effect was abolished. These findings are novel, as these studies represent first-in-human testing of Fer-1 pathways in human tissues, and specifically in human islets. These observations may be attributed to erastin’s ability to inhibit cystine uptake and contribute to subsequent GSH depletion^17^, as our study revealed that human islets exposed to erastin exhibited a significant increase in *CHAC1* expression relative to non-treated islets. Notably, *CHAC1* mRNA levels were unaffected by DFO or Fer-1 pre-treatment (data not shown) suggesting that glutathione depletion precedes the beneficial effects of these cytoprotective agents. CHAC1 expression levels may also provide a tool to differentiate between type 1 and type 2 ferroptosis induction alongside with determination of the intracellular GSH content. Prior studies have implicated GSH as a crucial antioxidant alleviating oxidative stress in islets. Glutamine, a precursor to GSH has previously been shown to enhance insulin secretion in response to glucose as well as reduce lipid peroxidation levels^32–34^. Miwa and colleagues^27^ demonstrated that islets treated with various lipid peroxidation products inhibited glucose-induced insulin secretion. Ample evidence suggests that when elevated in β-cells, fatty acids impair insulin gene expression, glucose-stimulated insulin secretion, and increase cell death^35^. It remains to be determined in more detail if these fatty acids also contain polyunsaturated fatty acids that were recently demonstrated to contribute to ferroptosis, dependent on the molecule PEBP1. However, little is known about ferroptosis in human tissues.

In our study, human islets pre-treated with Fer-1 and subsequently challenged with erastin exhibited improved viability and function than islets treated with erastin. Fer-1’s ability to mitigate the deleterious events associated with ferroptosis has previously been demonstrated in various cellular models, including Huntington’s disease^25^, and likely accounts for improved islet viability. It is possible that impaired insulin secretion observed in our study was a result of downstream events associated with engagement of the Xc_-_ antiporter through erastin treatment. GPX4 is capable of reducing intracellular ROS and requires GSH as an essential enzymatic co-factor^19^. Erastin treatment has previously demonstrated the ability to indirectly inhibit GPX4 activity by depleting GSH levels^20^. Koulajian et al.^28^ demonstrated improved in vitro and in vivo β-cell function in islets over-expressing GPX4 in the presence of lipid peroxidation products, further substantiating the necessity of GPX4 in preserving islet viability. On this premise, we sought to evaluate whether in vitro viability and function could indeed be compromised in the presence of the GPx4-specific inhibitor and FIA, RSL3. Given the importance of GPX4 in maintaining islet function and viability, increased levels of LDH indicate impaired viability. Islets challenged with RSL3 indeed revealed reduced viability, which was rescued in the presence of the ferroptosis inhibitor, Fer-1. However, the insulin-secreting capacity of islets exposed to 20 μM RSL3 exhibited only a modest insignificant decrease in sGSIS relative to non-treated islets and islets pre-conditioned with Fer-1. While this dose was capable of sufficiently inducing cell death, a higher dose of RSL3 may be required to confer impairment in insulin-secreting capacity, or regulated necrosis pathways other than ferroptosis (e.g., necroptosis or pyroptosis) may be involved.

Though treatment of human islets with both FIAs utilized in this study revealed compromised in vitro viability, pre-treatment of islets with erastin prior to transplant did not impair engraftment when transplanted under the renal capsule of immune-deficient recipients with a transplant dose (1500 IEQs). Early observations in the in vivo marginal study revealed both control islet recipients became euglycemic in parallel to all Fer-1-treated islet recipients. Control, Fer-1-and DFO-treated islets exhibited similar LDH levels in vitro, suggesting minimal cytotoxicity in culture without erastin or RSL3 treatment. An important explanation for differences between the positive impact of Fer-1 in vitro, but not seen in vivo, is that Fer-1 has a short half-life, and therefore may have had limited efficacy if ferroptosis was ongoing in vivo.

It is important to emphasize in the current study that human islets only became available for research study 24–72 h post isolation and subsequently assessed an additional 48 h in our experimental conditions. It is likely that any major negative contribution of ferroptosis could have already occurred within the unstable multi-organ donor, during prolonged cold ischemia, and the preceding islet isolation and culture periods. The impact we observed in vitro therefore reflects a secondary wave of inducible injury from the FIAs. This, coupled with the fact that we recounted and compensated for any numeric loss of human islets sustained during erastin or RSL3 secondary culture when we transplanted the human islets across groups, may explain why the dominant negative impact of FIAs was seen only in vitro but masked in vivo. The negative impact of both FIAs in vitro was relatively modest (~15–20% inducible cell death only, even at maximal doses). This further suggests that the FIAs are acting through non-dominant pathways, and that the majority of islets remain preserved. The present study therefore does not completely define the full potential of ferroptosis inhibition in islet transplantation, especially if this could be applied early in the multi-organ donor, and across all steps of transport, islet isolation and culture, which was not addressed herein.

Utilizing Fer-1 during islet isolation and acutely post culture may confer benefit to human islets and would be worth investigating in the experimental setting to determine its prospective clinical utility. It may also be necessary to evaluate the potential cytoprotective effect of Fer-1 using a more clinically relevant transplant site, like the hepatic portal vein. Our group previously revealed that the cytoprotective effects of the apoptosis inhibitor, F573, conferred varying engraftment outcomes dependent on the site of transplantation^36^. Moreover, this study also revealed the necessity to administer the drug to the organ donors and/or recipients could potentially confer improved engraftment outcomes. In this regard, administration of Fer-1 or other more potent ferroptosis inhibitors to donors or recipients may be necessary to confer maximal benefit to islet engraftment. More stable ferrostatins are currently being designed.

We and others, have suggested that Fer-1 exhibits lower in vivo stability, and have developed a third-generation ferroptosis inhibitor that exhibits improved plasma and metabolic stability^17^. Expanding the utility of new generation inhibitors of ferroptosis during islet isolation to prevent accumulated damage may be an attractive avenue to explore. The administration of ferrostatins as a co-therapy to islet transplant recipients or patients affected with type 2 diabetes may also yield improved clinical outcomes. Deterring early islet loss during isolation and subsequent clinical transplant is critical for long-term graft function. Utilizing novel cell death inhibitors to diminish islet damage in vitro and in the acute transplant period may be an attractive combination therapy to preserve islet mass and improve engraftment.

## Materials and methods

### Erastin, RSL3, Fer-1, and DFO

Erastin and Fer-1 (Sigma, Oakville, ON) were prepared by dissolving the drugs in phosphate buffered saline (PBS) at a stock concentration of 10 mM. DFO (Sigma, Oakville, ON) was prepared by dissolving the drug in water at a stock concentration of 75 mM. RSL3 was received by the Stockwell Laboratory (Columbia University, NY) and was prepared by dissolving the drug in dimethyl sulfoxide (DMSO) at a stock concentration of 40 mM. For long-term storage, both reagents were stored at −20 °C.

### Human islet isolation, purification, and culture

Human islet preparations were isolated after family consent to retrieve pancreas organs from deceased multi-organ donors, as previously described^37^, with intent for clinical transplantation and were only made available for research when the islet yield fell below that of the minimal mass required. Permission regarding the performance of these studies was granted by the Health Research Ethics Board at the University of Alberta (Edmonton, Alberta, Canada), after written permission from donor families. Human islets were cultured in clinical grade CMRL-1066 media (Media Tech, MT99-603-L) supplemented with insulin selenium-transferrin and insulin-like growth factor-1 at 22 °C and were received 24–72 h after isolation.

### Islet culture

Human islets were cultured in Connaught Medical Research Laboratories (CMRL-1066) medium supplemented with 10% fetal bovine serum, l-glutamine (2 mM), penicillin (50,000 units), streptomycin (50 mg), HEPES (5 mM), nicotinamide (10 mM), and sodium pyruvate (5 mM). For select erastin experiments, islets were maintained in CMRL ± 1, 5 or 10 μM of the ferroptosis inhibitor Fer-1 (Sigma, Oakville, ON) for 24 h. Subsequently, islets were collected, quantified, and cultured in the above conditions ±20 or 50 μM FIA, erastin (Sigma, Oakville, ON), for an additional 24 h. For RSL3 experiments, islets were maintained in CMRL ± 1 μM Fer-1 for 24 h and subsequently cultured in the aforementioned conditions ±20 μM RSL3.

### Lactate dehydrogenase as a measure of cytotoxicity

Human islets were cultured in the aforementioned conditions in non-tissue treated six-well plates (Costar, Corning, NY). Cell-free supernatants were subsequently collected and assessed for LDH release with the Cytotoxicity Detection Kit (Roche). Percentage cytotoxicity was calculated as per the manufacturer’s protocol using the formula: (test LDH release − spontaneous release)/maximal release. Test LDH release is the LDH released after treatment with the various treatment conditions; spontaneous release is the baseline cell LDH release; and maximal LDH release is the release of LDH when cells are lysed with 5% Triton-X. The data are the means of at least three independent experiments ± SEM.

### Static glucose-stimulated insulin secretion

Subsequent to 24-h culture, islets were collected from control (CMRL only) and treatment groups and were subjected to static GSIS (sGSIS). For each experiment, 50 islet equivalents (IEQs) from each group were incubated in RPMI-1640 containing low (2.8 mmol/L) glucose for 1 h, followed by high (16.7 mmol/L) glucose for an additional hour. Subsequent to glucose challenge, cell-free supernatants were collected and insulin levels were measured by enzyme-linked immunosorbent assay (Mercodia, Uppsala, Sweden). Stimulation index is represented as the ratio of insulin secreted in response to high glucose vs. insulin secreted in response to low glucose.

### Quantitative real-time PCR

RNA was extracted from islet preparations post culture (RNeasy Mini Kit; QIAGEN). Complementary DNA was synthesized by using the High-Capacity Reverse Transcription Kit (Thermo Fisher Scientific), and relative quantification (RQ) was performed by using TaqMan Gene Expression Assays (Thermo Fisher Scientific) with SDS software on the ABI PRISM 7900HT. Validated primer sets were as follows: PTGS2 (Hs00153133_m1), CHAC1 (Hs00225520_m1), and GAPDH (Hs02786624_g1), a housekeeping gene. Analysis by RQ software (ABI 7900HT) used the ΔΔCt method, and data were plotted as RQ.

### Diabetes induction and islet transplantation

One week prior to transplantation, immunodeficient C57BL/6 RAG^−/−^ mice (Jackson Laboratories, Bar Harbor, ME, USA) 12–14 weeks of age were rendered diabetic by chemical induction with intraperitoneal streptozotocin (STZ) (Sigma-Aldrich Canada Co., Oakville, ON, Canada), at 185 mg/kg in acetate phosphate buffer, pH 4.5. Diabetes was confirmed when non-fasting blood glucose levels exceeded 15 mmol/L for 2 consecutive daily readings.

For marginal human islet transplants, 24 h post culture ± 1 μM Fer-1 islets were quantified and transplanted under the kidney capsule at a dose of 500 IEQs ± 10% per diabetic recipient. For full-dose human islet transplants, islets were cultured for 24 h ± 1 μM Fer-1 and an additional 24 h ± 50 μM erastin, were collected, quantified, and transplanted under the kidney capsule at a dose of 1500 IEQs ± 10% per diabetic recipient. From the time of islet isolation to full-dose islet transplantation, the median culture period was 96 h.

For all transplants, human islets were aspirated into polyethylene (PE-90) tubing using a micro-syringe, and centrifuged into a pellet suitable for transplantation. A left lateral paralumbar incision was made and the left kidney delivered. The renal capsule was incised and the islets were infused.

### Evaluation of islet graft function

Non-fasting blood glucose measurements (mmol/L) were assessed three times weekly using a portable glucometer (FreeStyle InsuLinx, Abbott Diabetes Care Ltd., Oxon, UK) in the three transplant groups tested. Graft function and reversal of diabetes was defined as two consecutive readings ≤11.1 mmol/L and maintained until study completion. IPGTTs were conducted at study endpoint; 35 days post transplant for marginal islet recipients and 40 days post transplant in full-dose islet transplant recipients. Mice were fasted overnight prior to receiving an intraperitoneal 25% glucose bolus (3 g/kg). Blood glucose levels were evaluated at baseline (time 0), 15, 30, 60, 90, and 120 min post injection. Blood glucose AUC was calculated and analyzed between transplant groups.

### Islet graft retrieval

In order to corroborate graft-dependent euglycemia, islet transplants were retrieved by recovery nephrectomy. Islet transplant recipients were placed under anesthesia, and their graft-bearing kidney was exposed. Using a LT200 Ligaclip (Johnson & Johnson, Inc., Ville St-Laurent, QC, CA), the renal vessels and ureter were ligated and the islet graft-bearing kidney was removed. Non-fasting blood glucose measurements were monitored up to 7 days post graft removal to confirm hyperglycemia and thus post-transplant graft function.

### Statistical analysis

All data are represented as the mean ± SEM Islet viability data comparisons between control and treatment groups were analyzed through parametric one-way analysis of variance (ANOVA). Tukey’s post-hoc tests were used following the analysis of variances for multiple comparisons between study groups. IPGTT AUC was also analyzed by parametric one-way ANOVA. Data was analyzed using GraphPad Prism (GraphPad Software, La Jolla, CA, USA). *p* < 0.05 was considered significant.
